# Advanced Basal Cell Carcinoma: Epidemiology and Therapeutic Innovations

**DOI:** 10.1007/s13671-014-0069-y

**Published:** 2014-02-09

**Authors:** Shalini V. Mohan, Anne Lynn S. Chang

**Affiliations:** Department of Dermatology, Stanford University School of Medicine, 450 Broadway St, MC 5334, Pavilion C, 2nd floor, Redwood City, CA 94063 USA

**Keywords:** Basal cell carcinoma, Advanced basal cell carcinoma, Metastatic basal cell carcinoma, Smoothened inhibitors, Hedgehog pathway inhibitors, Vismodegib, Targeted therapy, Basal cell nevus syndrome, Gorlin’s syndrome

## Abstract

Advanced basal cell carcinomas are a subset of basal cell carcinomas that can be difficult to treat either due to their local invasiveness, proximity to vital structures, or metastasis. The incidence of all basal cell carcinoma is increasing in the United States, although it is not known whether advanced basal cell carcinomas (aBCCs) are also increasing. Recently, highly targeted therapy based on knowledge of the basal cell carcinoma pathogenesis has become available either commercially or through human clinical trials. These orally available drugs inhibit the Hedgehog signaling pathway, and lead to advanced basal cell carcinoma shrinkage that can enable preservation of adjacent vital organs. In this review, we outline the role of Hedgehog pathway inhibitors as well as other treatment modalities such as excision, radiotherapy and more traditional chemotherapy in treating advanced basal cell carcinomas. We also highlight current gaps in knowledge regarding the use and side effects of this targeted therapy.

## Introduction

The recent introduction of targeted therapy for advanced basal cell carcinomas (aBCCs) in the form of Hedgehog signaling pathway inhibitors represents one of the greatest triumphs of translational medicine, bridging basic science of a conserved developmental pathway with clinical application in patients with difficult-to-treat skin cancers [1, 2, 3, 4, 5]. In the coming years, the number of agents in this drug class is expected to increase. These drugs will be a powerful tool to complement, or in some cases substitute, for traditional treatment modalities for aBCCS, such as excision or radiotherapy.

## Epidemiology

BCC comprises the majority of non-melanoma skin cancers (NMSCs) and is more common than all other human malignancies combined. Several lines of evidence suggest that the worldwide incidence of BCCs is increasing. In the United States, the diagnosis and treatment of NMSCs has increased dramatically with a growth rate of 77 % over the past two decades. The fastest growing group is in women under the age of 40 years [6, 7]. In other countries such as Singapore where incidence of BCCs is monitored, the rate of BCCs has been rising over the past several decades as well [8]. Overall, the reasons for this dramatic growth have been postulated to include the aging population, changes in sun exposure habits, environmental changes, migration patterns, and to a lesser extent, increased prevalence of immunosuppressant use [9, 10].

More than 2.8 million new cases of BCC are diagnosed each year in the United States alone, and are estimated to result in over 3,000 deaths [1]. Fortunately, BCCs are usually diagnosed early and treated [11]. Nevertheless, a recent large, retrospective analysis from a major academic center reported 5-year recurrence rates at 2–3 % [12]. Given the high incidence of BCCs, this recurrence rate results in a large number of BCCs that are not cured by surgical excision. Furthermore, BCCs that are extensive and infiltrate structures below the skin or abut vital structures such as the brain or eyes may be difficult to surgically clear without significant morbidity. Many of these may become locally advanced or metastasize. BCCs that metastasize to either local or distant lymph nodes or distant organs would best be addressed through systemic therapy. Together, these locally advanced or metastatic BCCs comprise a disease group termed “advanced BCCs” (aBCCs).

While accurate estimates of the incidence of aBCCs are difficult to obtain, in part due to the lack of widespread use of a staging system by dermatologists and lack of uniform reporting requirements for NMSCs, aBCCs are thought to represent roughly 1–10 % of all BCCs, with metastatic BCCs accounting for 0.0028–0.5 % [13–15]. From our clinical experience, patients presenting with aBCCs appear to fall into two categories, (1) those who present with aBCC due to delay in accessing medical attention; or (2) those who have BCCs that are intrinsically aggressive and are refractory or recur after treatment.

## Economic, Physical and Psychosocial Impact of Advanced BCCs

NMSCs including BCCs account for 4.5 % of Medicare cancer expenditures, making it the fifth most costly cancer to treat [16, 17]. From 2005 to 2008, NMSCs accounted for $2.9 billion in average annual expenditures on cancer conditions among adults in the U.S. While aBCCs are uncommon, their size and location may require excision in the operating room with specialized surgical teams and postoperative hospital stays, likely leading to far greater medical costs than operable BCCs that can be managed in the office setting. When non-surgical modalities are utilized in aBCCs, such as radiation or chemotherapy, the cost of treating aBCCs rises dramatically, although there are no studies to date that have formally quantitated this. Therapeutic innovations such as Hedgehog pathway inhibitors may improve quality of life and extend the length of productive lifespan in some patients; however, this treatment has a significant financial cost, as a one-month supply of vismodegib currently exceeds $7,500. Patients who respond to the drug and tolerate side effects may be on this drug indefinitely, as currently there is insufficient data as to when this drug might be discontinued, heightening long-term costs.

Exploratory studies into the physical and psychosocial impact of advanced BCCs are underway; however, the disease can be challenging to characterize, in part due to variable progression and rarity of the disease [18, 19]. Anecdotal evidence from our clinical experience suggests that aBCC patients are subject to significant physical and psychological burden. Physical burdens including pain, blood loss with anemia and fatigue, infection risk with open wounds, limitations in movement or function due to location of aBCCs and side effects of surgery, radiation or chemotherapy. Psychosocial burdens from aBCC disease include depression, anxiety, social isolation, depleted financial resources from treatments, inability to find or maintain employment or inability to provide for or care for dependent family members. Even patients whose disease is stable or successfully treated can have significant limitation in functional ability due to scarring, disfigurement, and/or chronic pain.

Life expectancy in aBCC patients also contributes to the economic impact of this disease. Historically, reports of survival after diagnosis of distant metastasis in BCC patients were grim, estimated at 8–14 months [20]. A more recent retrospective case series from 1997–2011 suggested markedly improved survival in patients followed at a tertiary care center, with median survival time of 7 years [13]. Future analysis will be needed to identify factors that contribute to survival time, and the financial cost of any treatments associated with survival prolongation.

## Therapeutic Innovation of Advanced BCC Treatment

Historically, treatment options available to patient with aBCCs were not necessarily based on an understanding of the molecular characteristics of BCC pathogenesis. Treatment options included surgery, radiation, and traditional chemotherapies such as cisplatin-based treatment, but without systematic trials to follow for efficacy and safety, in part due to the rarity and heterogeneity of the condition (Table 1). In essence, there was no clearly established or superior treatment modality for aBCCs.

**Table 1:** Treatment modalities for advanced basal cell carcinoma (aBCC). Summary of therapeutic options, estimated response and recurrence rate and types of evidence

	Treatment modality	Type of aBCC	Response Rate*	Recurrence Rate	Type of Evidence in aBCCs	Possible Side Effects or Limitations	References
Surgical	Excision with margin evaluation	Locally advanced	NA	0.7 % after 4 years in a study including high risk facial lesions	No high quality studies comparing excision with Moh’s surgery in locally advanced BCCs alone due to rarity of disease; one randomized study included periocular BCCs with no difference in recurrence rate in excision (*n* = 5) versus Moh’s (*n* = 6)	incomplete removal, scarring, poor functional or cosmetic outcome	[12, 38–41]
	Moh’s micrographic surgery	Locally advanced	NA	NA but likely less than excision		incomplete removal, skip areas	
Field Therapy	Radiotherapy	Locally advanced	NA	7.5 % after 4 years in study including high risk facial lesions	Randomized trial comparing radiotherapy with surgery including high risk lesions	Less cosmesis than surgical treatment, scarring and dyspigmentation, risk of secondary cancer	[41, 42]
Targeted chemotherapy	Smoothened inhibitor (vismodegib)	Locally advanced; metastatic	30–43 %	20 %	Cohort studies for response rate; small retrospective study for recurrence rate	Muscle spasms, dysgeusia, alopecia, fatigue, nausea	[30••, 43, 44]
Non-targeted chemotherapy	Cisplatin-based chemotherapy	Locally advanced; metastatic	71 %	-	Case series of seven BCC patients with locally advanced disease using cisplatin and doxorubicin; case reports of cisplatin leading to complete response in a few metastatic patients	Severe nausea and vomiting, diarrhea, alopecia, joint pain, loss of balance, tinnitus, edema, fatigue	[45–49]

* Estimated response rate based on range derived from cited references. Please see original articles for specific details and explanation for basis of estimate

**Fig. 1:**
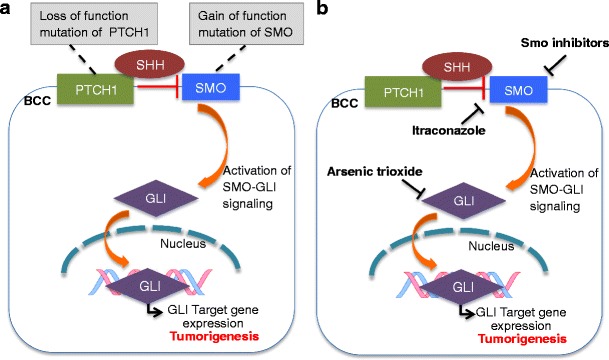
Simplified schematic of common mutations in the Hedgehog signaling pathway leading to basal cell carcinoma from pathway activation (**a**), and therapeutic targets of this pathway (**b**)

In the 1990s, the connection between aberrations of the Hedgehog signaling pathway and BCCs in mice was made [21•]. Around the same time, multiple studies connected this pathway in humans with both sporadic BCCs and an autosomal dominant genetic syndrome predisposing to multiple BCCs, basal cell nevus syndrome (BCNS) or Gorlin-Goltz syndrome [22]. The majority of mutations in sporadic BCCs and BCNS patients occur in *PTCH1* [23, 24], a protein that inhibits Smoothened. BCNS patients experience early onset and numerous BCCs, due to loss-of-heterozygosity in the PTCH1 gene. The second most common mutation in sporadic BCCs and BCNS patients are gain-of-function mutations of the *Smoothened* gene [25, 26]. Loss of *PTCH1* results in the lack of Smoothened inhibition, leading to increases in *GLI1* levels, changes in transcription, and subsequent tumorigenesis. Gain-of-function *Smoothened* mutations also leads to increased *GLI1* levels and tumorigenesis [27]. A simplified schematic of the Hedgehog (Hh) pathway is presented in Fig. 1a.

The story of how the first inhibitor of the Hh pathway, cyclopamine, was discovered is one of the most fascinating in biomedicine. In the 1960s, pregnant ewes ingesting the California corn lily were found to produce one-eyed offspring, or extreme holoprosencephaly [28]. In the 1970s, the active agent inducing these changes, cyclopamine, was isolated and its structural formula identified. Subsequent studies in the 1990s in chick embryos demonstrated cyclopamine’s ability to induce holoprosencephaly and to bind the transmembrane protein, Smoothened [29]. Subsequently, a number of analogues were developed by modifications to cyclopamine to improve solubility, and oral bioavailability. Collectively, these analogues are called Smoothened inhibitors (SIs), due to their targeting of the Smoothened protein (Fig. 1b).

Multiple orally available SIs are currently in human clinical trials against BCCs. The first and only U.S. Food and Drug Administration (FDA) approved SI for aBCCs to date is vismodegib, which became commercially available in 2012. The phase II study with 96 aBCC patients leading to FDA approval demonstrated an independently assessed response rate of 30 % in patients with metastatic BCC and 43 % response rate in locally advanced BCC [30••]. A subsequent study of 119 aBCC patients showed similar findings [31]. The availability of vismodegib as highly targeted therapy for aBCCs is one of the greatest success stories in translational medicine.

While most SIs in human trials come from the research pipelines of pharmaceutical companies, two existing FDA approved drugs have shown activity against the Hh pathway. These are oral ketoconazole and intravenous arsenic trioxide, both of which are being been tested in mouse models [32•] and a small number of BCC patients in the research setting, with results currently unpublished.

## Role of Smoothened inhibitors in Advanced BCC Treatment

**Fig. 2:**
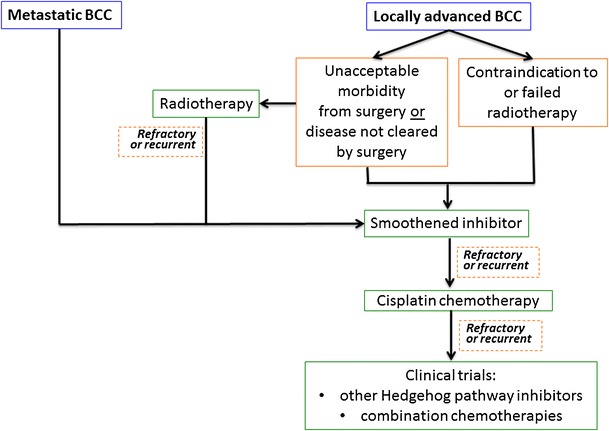
Example treatment algorithm for advanced basal cell carcinoma. Due to disease heterogeneity in advanced basal cell carcinomas, actual treatment depends on tumor location, prior treatments and patient comorbidities

Along with innovation in therapy come many questions that remain to be answered. First, which aBCC patients are appropriate for SI treatment? An example clinical decision tree is shown in Fig. 2. However, given the heterogeneity of aBCC patients as far as tumor location and extent and comorbidities, each patient should be considered on a case-by-case basis, in conjunction with multidisciplinary consultation such as medical oncology, radiation oncology and surgical specialties. In addition, patients may have differential tolerance for side effects of SIs. As a class, these side effects include muscle spasms, taste disturbance, alopecia, nausea, and fatigue [33]. Anecdotally, muscle spasms have been ameliorated with muscle relaxants such as cyclobenzaprine. Nausea and poor oral intake have been addressed with megestrol acetate or dronabinol. SIs are also potent teratogens and two forms of medically reliable birth control should be used in patients (and their partners) with reproductive potential. Patients on SI treatment need to follow closely with their treating physicians to monitor for tumor response and side effects. In addition to regular skin checks, patients may need regular radiologic imaging to monitor for disease recurrence in cases where the aBCC has a deep component or is metastatic to non-skin organs. If the aBCC is refractory or recurrent, timely intervention with other treatment modalities is critical.

Second, can the response rate for aBCCs be increased when combined with other treatment modalities, such as other chemotherapies or radiation treatment? Can recurrence rates be decreased when combined with other treatment modalities? Clinical trials are underway to explore these questions.

Third, what is the impact of SI treatment on progression free survival or overall survival in aBCCs? Given the rarity of this disease, multicenter registry studies are in progress to assess these.

Fourth, what is the impact of SI treatment on quality of life and psychosocial outcomes? The impact of SI treatment relates to the efficacy of the drug against the aBCC, as well as the tolerability of the side effects. Compared to many other forms of chemotherapy, the side effects of SI may be more tolerable. Alternatively, muscle spasms or other side effects may limit the ability of patients to take the drug in the long term, and/or maintain a good quality of life despite disease stabilization. Patient-based instruments recently have been developed to better assess these endpoints [18, 19].

Fifth, are there clinical predictors for response to SI treatment? One analysis from an expanded access study in patients for vismodegib identified prior treatment with systemic therapy as negatively correlated with laBCC response in 56 patients [34]. There was no association with age, prior radiotherapy or number of sites affected with aBCCs. In this same study, analysis of 39 metastatic BCCs showed that neither age, prior radiotherapy, prior systemic therapy or number of sites affected with aBCCs were associated with tumor response. Larger prospective studies are underway to assess clinical predictors for SI response.

Sixth, are there tumor markers from biopsies to predict response to SI treatment? Given the aggressiveness of many aBCCs, the decision of which treatment modality to undertake could be life-saving. Current studies are underway to assess for mutations, gene expression changes or protein levels within the Hh signaling pathway [35–37] that could correlate with response or lack of response, with the ultimate goal of assisting with clinical decision-making. As the current cost of SI drugs is quite high, a diagnostic test that could indicate the likelihood of response to these drugs may be cost effective.

Many additional issues remaining to be clarified regarding the use of SIs, including: effective management of common side effects, long-term side effects such as the potential for secondary cancers, whether there is differential response based on BCC subtype, when SI treatment may be safely discontinued after apparent complete clinical response, and whether SIs should be used to treat microscopic disease in surgical cases where margins are positive.

## Conclusion

Advanced BCCs are often difficult to treat and life-threatening. SI drugs can be life-saving in many cases, though disease progression or recurrence is a major concern that requires regular monitoring through skin examinations and/or radiologic imaging. Successful management of side effects is critical to continued treatment with these drugs. When aBCCs are refractory, achieve a partial response, or are recurrent after SI therapy, other treatment modalities such as radiation, traditional chemotherapy or surgical excision need to be considered for optimum patient outcomes.
